# Supplementary material for: Chromothripsis during telomere crisis is independent of NHEJ, and consistent with a replicative origin
Source: Genome Res. 2019 May;29(5):737–49. doi: 10.1101/gr.240705.118 (PMC6499312; doi:10.1101/gr.240705.118)
Supplement: Supplemental Material [file supp_gr.240705.118_Supplemental_file_1.zip › contigs/annotated_contigs/DB79/contig.2.DB79_length_402_mean_cov_7.08955223881.docx]

**DB79_length_402_mean_cov_7.08955223881**

```
AAGTTGGGATTATATAGCTAGGCCTTTATGACTCTGCATCAATCAGTCGTTGGTTAAGGAGTGTAACGTTAGACAAGAAAAGTCCTCAG
 >chr7:7081071-7081328 + E=2e-143
CTGAAAGCAGTGAATGGAGGGCTCTCAACCAGCAGTACTCGGATATGGCTGGTGCACCACACCAGCCTATCCTATGCAAAATGGCATAG

TTCCCTCCAATAAGTTCCTGTTATAACAGTTCCCTGATCCCCACTCTTCCTGGAGCCACTGACAGCTAACAGCAATAA|A|CTTGACTG
                                                                               >chr7:11
AAGGGAAAAATGGAAGATGGTAGACATCCTCTCAAGTGGATGTAGGAGGTAATTAGAATAATAAAATTGAATAATTATTAAGTAGTTTG
4641092-114641238 + E=2e-76
CTCAAGGATATTAATCATTCTGCCCATTTTGAAGGAGAAAAAATATTC
```
